# Gingerol prevents prion protein-mediated neuronal toxicity by regulating HIF prolyl hydroxylase 2 and prion protein

**DOI:** 10.3892/ijmm.2014.1936

**Published:** 2014-09-16

**Authors:** YANG-GYU PARK, SANG-YOUEL PARK

**Affiliations:** Biosafety Research Institute, College of Veterinary Medicine, Chonbuk National University, Jeonju, Jeonbuk 561-756, Republic of Korea

**Keywords:** gingerol, prion protein 106-126, prion diseases, hypoxia-inducible factor 1α, prolyl hydroxylase 2

## Abstract

Prion diseases are a family of progressive neurodegenerative disorders, which are fatal in the majority of cases and affect both humans and domestic animals. Prion protein (PrP) (106–126) retains the neurotoxic properties of the entire pathological PrPsc and it is generally used as a reasonable model to study the mechanisms responsible for prion diseases. In our previous studies, we demonstrated that hypoxia-inducible factor (HIF)-1α is involved in the gingerol-mediated protection of neuronal cells. HIF mediates cellular adaptations to low oxygen. Prolyl hydroxylase domain-containing protein 2 (PHD2) is an oxygen sensor that hydroxylates the HIF-α-subunit, promoting its proteasomal degradation under normoxic conditions. Thus, in the present study we wished to determine whether gingerol inhibits the catalytic activity of PHD2 and prevents HIF-1α protein proteasomal degradation, thereby preventing the occurrence of PrP (106–126)-induced neuronal apoptosis. We used the pharmacological inhibition of PHD2 by dimethyloxalylglycine (DMOG) or deferoxamine (DFO) and the genetic inhibition of HIF-1α by HIF-1α small interfering RNA (siRNA) to block the effects of gingerol against PrP (106–126)-induced neurotoxicity. Our results demonstrated that gingerol prevented PrP (106–126)-induced neuronal apoptosis by upregulating HIF-1α and inhibiting the catalytic activity of PHD2 under normoxic conditions. Moreover, the protective effects of gingerol against PrP (106–126)-induced neuronal apoptosis were associated with the upregulation of the expression of cellular prion protein (PrPc). In conclusion, our results indicate that gingerol has therapeutic potential for use in the treatment or prevention of prion diseases, and its inhibitory effects on the catalytic activity of PHD2 may be of clinical benefit.

## Introduction

Prion diseases are a family of progressive neurodegenerative disorders, which are fatal in the majority of cases and affect both humans and domestic animals. Prion diseaes are also termed as transmissible spongiform encephalopathy (TSE) and are characterized by the spongiform degeneration of the central nervous system (1). Prion diseases involve the conversion of normal cellular prion protein (PrPc) into the scrapie isoform of prion protein (PrPsc) (2). PrPsc is widely known as an infectious agent of prion diseases (3). In addition, it is more prone to be accumulated in insoluble fibrils and thereby disrupts normal neuronal functions (4).

The neuronal aggregation of PrPSc or neuronal cells exposed to prion protein (PrP) fragment [PrP (106–126)] induces mitochondrial malfunction, which has been reported to be major hallmark of neurodegenerative diseases, including Huntington’s disease and Alzheimer’s disease (5–9). A previous study demonstrated that treatment with gingerol prevents PrP (106–126)-mediated mitochondrial neurotoxicity through the regulation of hypoxia-inducible factor-1α (HIF-1α) activation (10). Xie *et al* (11) also demonstrated that the exposure of neurons to low oxygen activated HIF-1α and inhibited the activation of the mitochondrial apoptotic pathway induced by nerve growth factor (NGF) deprivation. These observations suggest that regulators of mitochondrial homeostasis, including HIF-1α, may be key factors for the protection against prion-related diseases.

A peptide corresponding to the residues 106–126 of the PrP sequence [PrP (106–126)] has been widely used to examine the mechanisms of prion-mediated neurotoxicity (12). Moreover, PrP (106–126) has been found to induce neuronal apoptosis in primary cultures of hippocampal, cortical and cerebellar neurons (13,14). Therefore, PrP (106–126) is a useful experimental model for *in vitro* studies of prion-induced neuronal apoptosis (15).

Typically, mammalian cells have the ability to recognize changes in the local availability of oxygen (16), which is a key element for cell survival. Under hypoxic conditions, whereby low oxygen levels are low, the hypoxic response pathway is activated. This leads to the increased levels of HIF-1 and its stabilization (17).

HIF-1 is a heterodimer that consists of α- and β-subunits (18). The α-subunit consists of 3 subunits, HIF-1α, HIF-2α and HIF-3α, all of which are structurally similar. Of these, HIF-1 is involved in the regulation of iron metabolism, angiogenesis and cell survival (19). Furthermore, in a previous study, we demonstrated that HIF-1α is involved in the regulation of the expression of prion protein to protect neurons (20). Under normoxic conditions, however, HIF-1α is degraded through the ubiquitin-proteasome pathway when the von Hippel-Lindau tumor suppressor protein (pVHL) binds to the oxygen degradation domain (ODD) mediated by the HIF prolyl hydroxylase domain-containing proteins (PHDs) (21). In mammalian cells, PHDs are composed of 3 isoforms, PHD1, PHD2 and PHD3, all of which have been shown to cause the hydroxylation of the key proline residues (Pro402 and Pro564) of HIF-1α in an *in vitro* setting (21,22). Furthermore, the HIF-1α protein can be stabilized by PHD enzyme inhibitors, such as deferoxamine (DFO) and dimethyloxalylglycine (DMOG) (23). Recent major advances have shown that HIF PHD2 is involved in the regulation of the ubiquitin-proteasome pathway associated with HIF-1α (3). Moreover, it is also a key oxygen sensor that creates low steady-state levels of HIF-1α under normoxic conditions (3).

Gingerol is the active constituent of fresh ginger, and it has been widely used as a Chinese herbal medicine in the treatment of a variety of diseases, including inflammation (24). Moreover, it is one of the pivotal bioactive products of ginger and has various pharmacological properties, such as anti-inflammatory and antioxidant activities. Furthermore, it is also involved in cell survival and neuroprotection (10,24,25). It has been reported that gingerol increases the protein levels of HIF-1α (10). However, little is known about the molecular mechanisms through which gingerol mediates the expression of HIF-1α in the pathogenesis of prion diseases.

Given the above background, we hypothesized that gingerol may inhibit the catalytic activity of PHD2 and thereby prevent the occurrence of PrP (106–126)-induced neuronal apoptosis through the upregulation of the protein expression of HIF-1α. To examine this hypothesis, in the present study, we investigated the effects of gingerol on PHD2 catalytic activity and assessed its role in the effects of HIF-1α on the occurrence of PrP (106–126)-induced neuronal apoptosis.

## Materials and methods

### Cell culture

The SH-SY5Y human neuroblastoma cell line was obtained from the American Type Culture Collection (ATCC, Rockville, MD, USA). The murine neuronal cell lines, ZW 13-2 and Zpl 3–4, established from the hippocampus of ICR (*Prnp*^+/+^) and Zürich I (*Prnp*^−/−^) mice, respectively, were kindly donated by Professor Yong-Sun Kim (Hallym University, Chuncheon, Korea). The SH-SY5Y cells were cultured in minimum essential medium (MEM; Invitrogen-Gibco, Grand Island, NY, USA), whereas the ZW 13-2 and Zpl 3–4 cells were cultured in Dulbecco’s modified Eagle’s medium (DMEM; HyClone, Logan, UT, USA) that contained 10% fetal bovine serum (FBS; Sigma-Aldrich, St. Louis, MO, USA) and penicillin-streptomycin (both 100 units/ml) in a humidified incubator maintained at 37°C and 5% CO_2_.

### Construction of HIF-1α short hairpin (sh)RNA plasmid

The shRNA against HIF-1α was kindly donated by Dr Yong-Nyun Kim (National Cancer Center, Goyang, Korea). The shRNA plasmid constructs for HIF-1α (shHIF-1α) were generated in the lentiviral vector, pL-UGIP. The shRNA for HIF-1α was obtained with the following oligonucleotide sequences: forward, 5′-CTGATGACCAGCAACTTGA-3′ and reverse, 5′-TCAA GTTGCTGGTCATCAG-3′. The SH-SY5Y cells were transfected with shHIF-1α, and stable transfectants were selected with puromycin after a 24-h recovery in standard growth medium. SH-SY5Y cells transfected with the mock vector (shMOCK) were used as the controls.

### Reagents

Gingerol (0.625, 1.25 and 2.5 nM) was purchased from Santa Cruz Biotechnology (Santa Cruz, CA, USA); DMOG (500 μM) and DFO (100 μM) and doxorubicin (DOX, 40 nM) were purchased from Sigma-Aldrich. In addition, cycloheximide (CHX, 50 μM) was purchased from Santa Cruz Biotechnology.

### PrP (106–126) treatment

The synthetic PrP (106–126) (sequence, Lys-Thr-Asn-Met-Lys-His-Met-Ala-Gly-Ala-Ala-Ala-Ala-Gly-Ala-Val-Val-Gly-Gly-Leu-Gly) was synthesized by Peptron Inc. (Daejon, Korea). The peptide was dissolved in sterile dimethyl sulfoxide (DMSO) at a concentration of 10 mM and then stored at −80°C.

### Western blot analysis

The SH-SY5Y cells were lysed in buffer containing 25 mM HEPES; pH 7.4, 100 mM NaCl, 1 mM EDTA, 5 mM MgCl_2_, 0.1 mM dithiothreitol (DTT) and protease inhibitor mixture. Proteins were electrophoretically resolved by 10–15% sodium dodecyl sulfate-polyacrylamide gel electrophoresis (SDS-PAGE), and immunoblotting was performed as previously described. Equal amounts of lysate protein were similarly electrophoretically resolved and electrophoretically transferred onto a nitrocellulose membrane. Immunoreactivity was detected through sequential incubation with horseradish peroxidase-conjugated secondary antibody and enhanced chemiluminescence reagents. Antibodies used for immunoblotting were HIF-1α (Pierce Biotechnology, Rockford, IL, USA), HO-HIF-1α (Cell Signaling Technology, Boston, MA, USA), PHD2 (Abcam, Cambridge, MA, USA), PrPc (Millipore, Billerica, MA, USA) and β-actin (Santa Cruz Biotechnology).

### Annexin V assay

Apoptosis was assessed using a commercial Annexin V assay (Santa Cruz Biotechnology) according to the manufacturer’s instructions using a flow cytometry method. The Annexin V content was determined by measuring fluorescence at an excitation wavelength of 488 nm and emission wavelengths of 525 and 530 nm using a Guava easyCyte™ flow cytometer (Millipore, Bedford, MA, USA).

### Immunofluorescence staining

The SH-SY5Y cells cultured on glass coverslips were treated with PrP (106–126). The cells were washed with phosphate-buffered saline (PBS) and fixed with cold acetone for 90 sec. The cells were then washed with PBS, blocked with 5% fetal bovine serum in Tris-buffered saline containing Tween-20, and incubated with anti-HO-HIF-1α (2 μg/ml) and PHD2 (2 μg/ml) and anti-HIF-1α (2 μg/ml) monoclonal antibodies for 48 h at 20°C. Unbound antibody was removed by an additional PBS wash, and the cells were incubated with labeled anti-rabbit Alexa Fluor^®^ 546 (for anti-HO-HIF-1α and PHD2) IgG antibody (4 μg/ml) and Alexa Fluor 488 (for HIF-1α) IgG antibody (4 μg/ml) for 2 h at 20°C. Finally, the cells were counterstained with DAPI (blue) and mounted with DakoCytomation fluorescent medium (Dako, Glostrup, Denmark) and visualized under a fluorescence microscope.

### RNA interference (RNAi)

The SH-SY5Y cells were transfected with HIF-1α small interfering RNA (siRNA; oligo ID HSS104775; Invitrogen, Carlsbad, CA, USA) using Lipofectamine™ 2000 according to the instructions of the manufacturer. Following culture for 48 h, the knockdown efficiency was typically measured at the protein level by an immunoblot assay.

### Reverse transcription quantitative polymerase chain reaction (RT-qPCR)

Total RNA was extracted from the SH-SY5Y cells using easy-spin™ Total RNA Extraction kits (Intron Biotechnology, Seoul, Korea). cDNA synthesis was carried out following the instructions provided with the Takara PrimeScript^®^ First Strand cDNA Synthesis kit (Takara Bio, Inc., Shiga, Japan). For RT-qPCR, 1 μl of gene primer with SYBR^®^-Green (Bio-Rad Laboratories, Hercules, CA, USA) in 20 μl of reaction volume was applied. The sequences of the primers used for the qPCR were as follows: HIF-1α forward, 5′-AGAAACCAC CTATGACCTGC-3′ and reverse, 5′-GTCGTGCTGAATAAT ACCACTC-3′; PHD2 forward, 5′-CAAGGACATCCGAGG CGATAAG-3′ and reverse, 5′-CCGTTACAGTGGCGTATC AGG-3′; and β-actin (as an internal control) forward, 5′-GCAA GCAGGAGTATGACGAG3′ and reverse, 5′-CAAATAAA GCCG CCAATC-3′. All reactions with iTaq™ SYBR-Green Supermix (Bio-Rad Laboratories) were performed using the CFX96™ real-time PCR detection system (from Bio-Rad Laboratories).

### Statistical analysis

All data are expressed as the means ± standard deviation (SD), and were compared using the Student’s t-test and ANOVA with the Duncan test using the SAS statistical package (SAS Institute, Inc., Cary, NC, USA). Statistical significance was set at P<0.05 or P<0.01.

## Results

### The gingerol-induced increase in the expression of HIF-1α prevents PrP (106–126)-induced neurotoxicity

**Figure 1:**
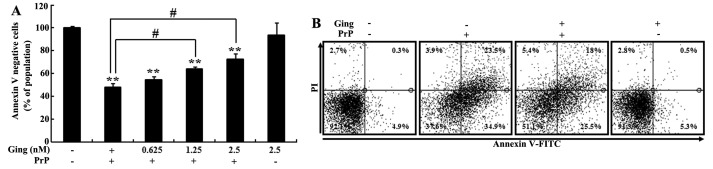

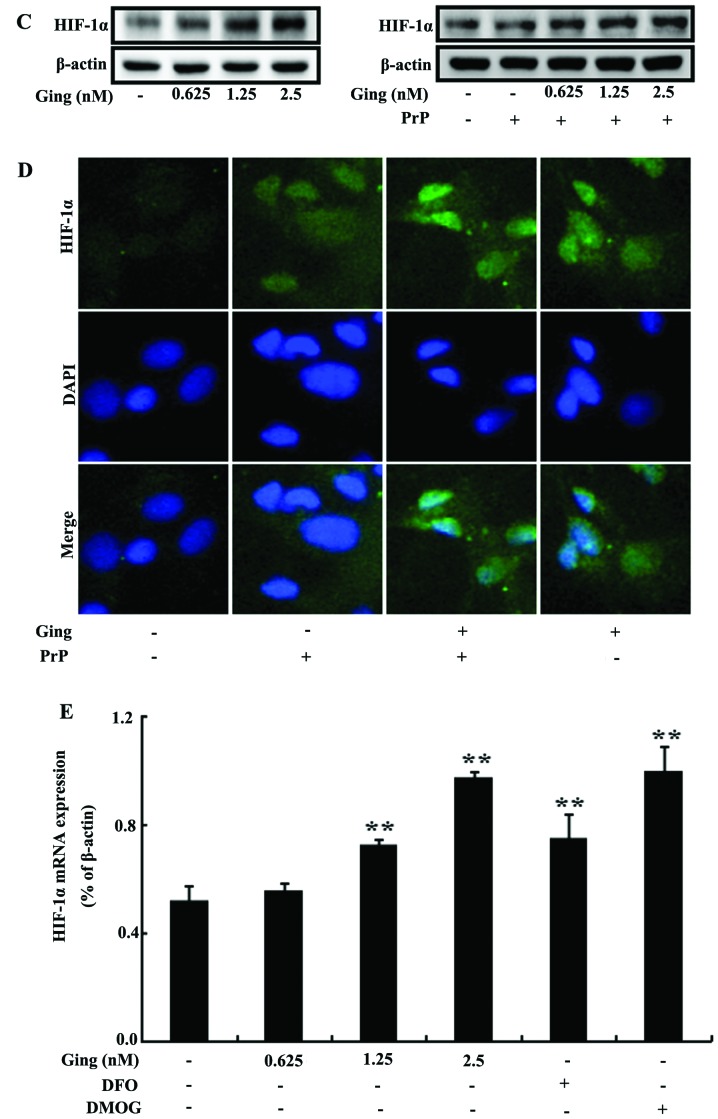
Gingerol regulates the expression of hypoxia-inducible factor (HIF)-1α under normoxic conditions and protects neuronal cells from PrP (106–126)-induced apoptosis. (A) SH-SY5Y neurons were pre-treated with various concentrations of gingerol for 12 h and then exposed to 100 μM PrP (106–126) for 12 h. Bar graph indicates that the average numbers (%) of Annexin V-negative cells. ^**^P<0.01 vs. controls (untreated cells), ^#^P<0.01 vs. PrP (106–126)-treated cells. (B) Cell viability was measured by an Annexin V assay and flow cytometry. SH-SY5Y neurons were pre-treated with 2.5 nM gingerol for 12 h and then exposed to PrP (106–126) 100 μM for 8 h. (C) The treated cells were assessed for the protein expression of HIF-1α by western blot analysis. The results were normalized to those of β-actin. (D) The treated cells were also assessed for the protein expression of HIF-1α by immunofluorescence staining. (E) HIF-1α mRNA levels were measured by RT-qPCR. The results are representative of 2 independent experiments. ^**^P<0.01 vs. controls. Ging, gingerol; DFO, deferoxamine; DMOG, dimethyloxalylglycine.

It has recently been demonstrated that the gingerol-induced expression of HIF-1α inhibits the occurrence of human prion peptide-mediated neurotoxicity (20). Based on this report, we reconfirmed the protective effects of gingerol on PrP (106–126)-induced neuronal apoptosis (Fig. 1). The SH-SY5Y cells were pre-treated for 12 h with various concentrations of gingerol and then exposed to 100 μM PrP (106–126) for 12 h (Fig. 1A). The Annexin V-negative cell population was decreased following treatment with PrP (106–126). Following pre-treatment with gingerol, however, there was an increase in the PrP (106–126)-induced Annexin V-negative SH-SY5Y neuronal cell population (Fig. 1A and B). These results indicate that gingerol prevents PrP (106–126)-induced neurotoxicity.

In our previous studies, we demonstrated that HIF-1α is involved in the regulation of the expression of prion protein to protect neurons (20,26). Indeed, gingerol increases the protein levels of HIF-1α (15). To determine whether HIF-1α is involved in the protective effects of gingerol, we assessed the protein and mRNA levels of HIF-1α in the SH-SY5Y cells (Fig. 1C–E). The SH-SY5Y cells were pre-treated with various concentrations of gingerol for 12 h (Fig. 1C) and then exposed to PrP (106–126) for 8 h. In the cells treated with gingerol, there was an increase in the protein levels of HIF-1α (Fig. 1C). These results were confirmed by immunofluorescence staining (Fig. 1D). To determine whether gingerol is involved in the regulation of the expression of the HIF-1α gene, the SH-SY5Y cells were incubated for 24 h in the presence of gingerol. The expression of HIF-1α gene was then measured by RT-qPCR. Following treatment with gingerol, there was a dose-dependent increase in the mRNA expression of HIF-1α (Fig. 1E).

**Figure 2:**
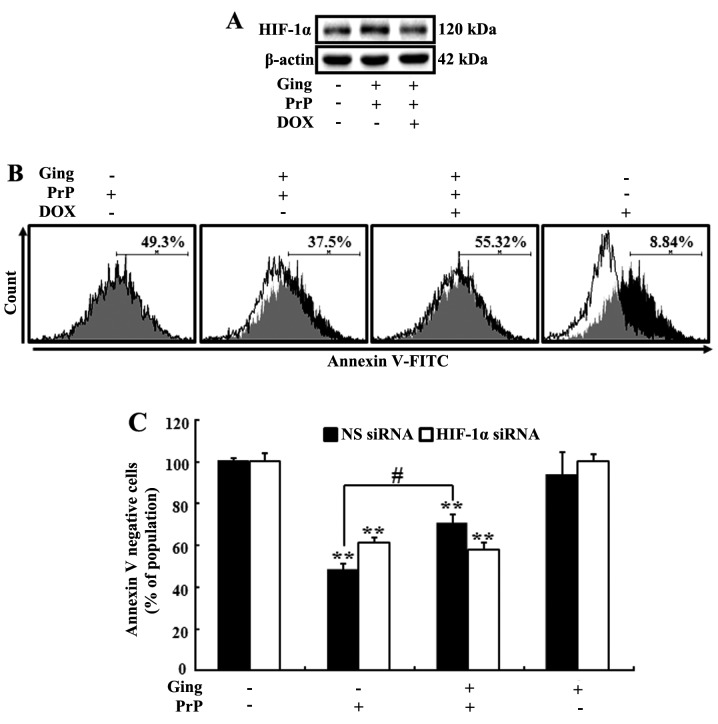

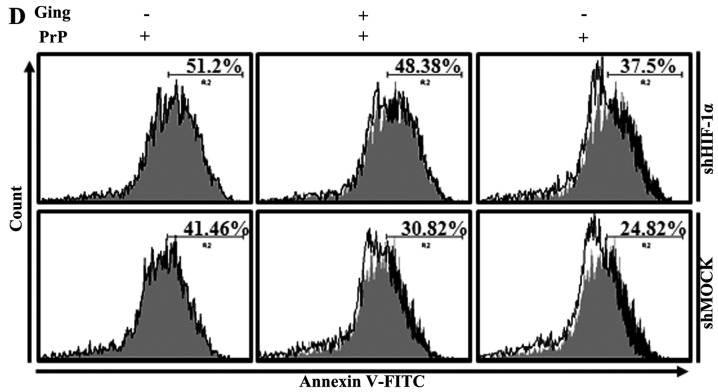
Gingerol prevents the occurrence of PrP (106–126)-induced neuronal apoptosis by inhibiting the expression of hypoxia-inducible factor (HIF)-1α. (A) SH-SY5Y cells were pre-treated with 2.5 nM gingerol for 12 h and/or 40 nM doxorubicin (DOX) for 1 h prior to treatment with gingerol. The cells were then exposed to 100 μM PrP (106–126) for 12 h. The treated cells were assessed for the protein expression of HIF-1α by western blot analysis. The results were normalized to those β-actin. (B) The treated cells also were assessed for cell viability by an Annexin V assay and flow cytometry. (C) SH-SY5Y cells transfected with HIF-1α siRNA (HIF-1α small interfering RNA) or NS-siRNA (non-specific siRNA) were incubated with 100 μM PrP (106–126) for 12 h following exposure to 2.5 nM gingerol for 12 h. Cell viability was measured by an Annexin V assay and flow cytometry. Bar graph indicates the average number (%) of Annexin V-negative cells. ^**^P<0.01 vs. controls (untreated cells), ^#^P<0.01 vs. PrP (106–126)-treated cells. (D) HIF-1α shRNA or mock-transfected SH-SY5Y cells were treated with 2.5 nM gingerol for 12 h and then exposed to 100 μM PrP (106–126) for 12 h. The viability of the treated cells was assessed by an Annexin V assay and flow cytometry. Ging, gingerol; DOX, doxorubicin.

We used DOX as an HIF-1α inhibitor in order to examine the effects of gingerol in preventing the occurrence of PrP (106–126)-induced neurotoxicity through HIF-1α. The SH-SY5Y cells were incubated with PrP (106–126) for 8 h following exposure to 2.5 nM of gingerol (12 h) and/or 40 nM of DOX for 1 h prior to treatment with gingerol. As shown in Fig. 2A, the protein levels of HIF-1α were increased following treatment with gingerol. However, DOX inhibited the effects of gingerol on the protein levels of HIF-1α. Moreover, DOX inhibited the effects of gingerol on PrP (106–126)-induced neurotoxicity in the SH-SY5Y cells (Fig. 2B). These results were confirmed by RNAi experiments using Annexin V assay. As shown in Fig. 2C, gingerol prevented the occurrence of PrP (106–126)-induced neurotoxicity in the cells transfected with non-specific siRNA (NS siRNA), whereas treatment with gingerol had no effect on PrP (106–126)-induced neurotoxicity in the cells transfected with HIF-1α siRNA. These results were confirmed by shRNA experiments using the Annexin V assay for cell viability (Fig. 2D).

### Gingerol promotes the stabilization of HIF-1α through the inhibition of HIF PHD2 catalytic activity

**Figure 3:**
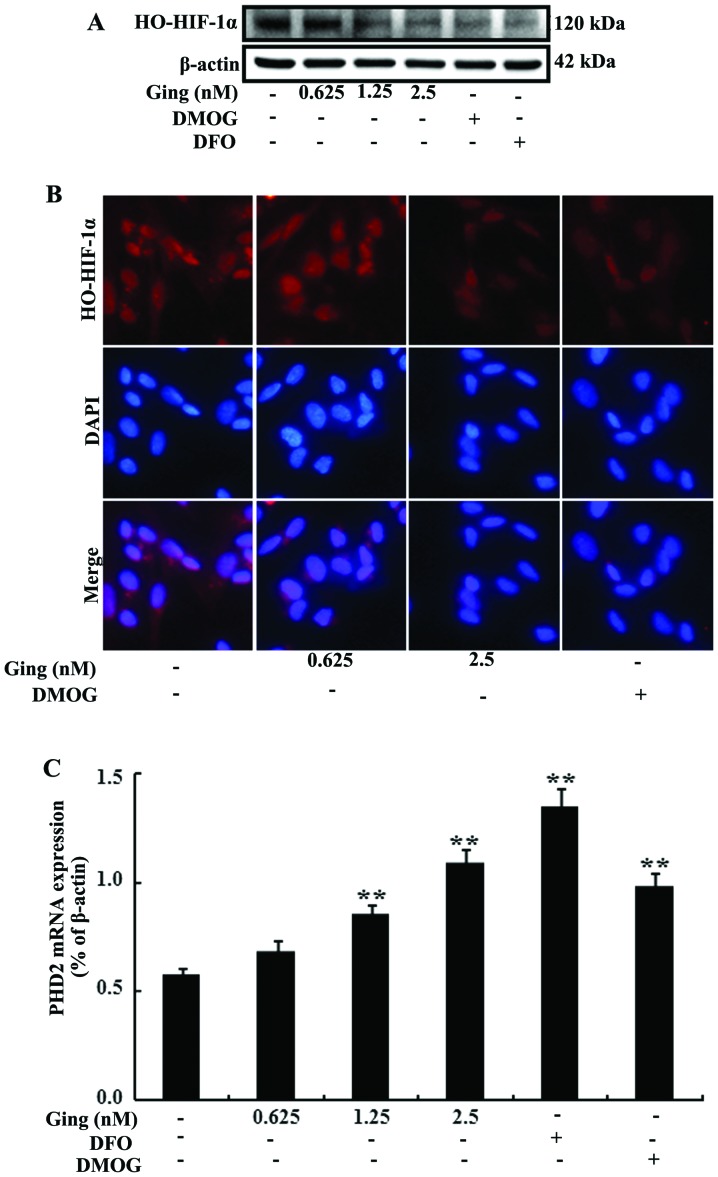

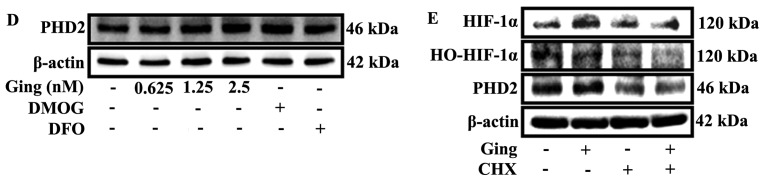
Gingerol inhibits the hydroxylation of hypoxia-inducible factor (HIF)-1α by inhibiting the expression of prolyl hydroxylase domain-containing protein 2 (PHD2). (A) SH-SY5Y cells were treated with 2.5 nM gingerol for 20 h, DMOG for 20 h or deferoxamine for 20 h. The treated cells were assessed for the protein expression of HO-HIF-1α by western blot analysis. (B) Protein expression was also analyzed by immunofluorescence staining. (C) SH-SY5Y cells were treated with various concentrations of gingerol for 24 h, DMOG for 20 h or deferoxamine for 20 h. HIF PHD2 mRNA levels were measured by RT-qPCR. ^**^P<0.01 vs. controls (untreated cells). (D) The treated cells also were assessed for the protein expression of PHD2 by western blot analysis. (E) SH-SY5Y cells were incubated with 100 μM PrP (106–126) for 8 h following exposure to 2.5 nM gingerol for 12 h and/or 50 μM cycloheximide for 1 h prior to treatment with gingerol, which was followed by the assessment of the protein expression of HIF-1α, HO-HIF-1α and PHD2 by western blot analysis. The results were normalized to those of β-actin (D and E). Ging, gingerol; DFO, deferoxamine; DMOG, dimethyloxalylglycine.

Under normoxic conditions, HIF-1α is degraded by catalytically activated PHD2 (2). Furthermore, the molecular mechanisms responsible for the gingerol-mediated induction of HIF-1α expression are not yet fully understood. In the present study, in order to examine the effects of gingerol on the hydroxylation of HIF-1α, the SH-SY5Y cells were treated with various concentrations of gingerol, and DMOG 500 μM or DFO (PHD enzyme inhibitors). As shown in Fig. 3A, following treatment with gingerol, there was a dose-dependent decrease in HIF-1α hydroxylation. Consistent with these results, immunofluorescence staining also revealed that HIF-1α hydroxylation was inhibited following treatment with gingerol (Fig. 3B). Furthermore, to determine whether gingerol is involved in the regulation of the mRNA expression of PHD2, the SH-SY5Y cells were incubated for 24 h in the presence of gingerol, DMOG or DFO. Subsequently, the mRNA expression of PHD2 was measured by RT-qPCR. The mRNA expression of PHD2 was increased in the SH-SY5Y cells following treatment with gingerol (Fig. 3C). In addition, following treatment with gingerol, there was an increase in the protein levels of PHD2 (Fig. 3D). We then assessed whether the gingerol-induced upregulation of PHD2 is related to HIF-1α stabilization by using CHX. For this purpose, the SH-SY5Y cells were incubated with PrP (106–126) for 8 h following exposure to 2.5 nM gingerol (12 h) and/or 50 μM CHX for 1 h prior to treatment with gingerol. In the CHX-treated group, there was a decrease in the protein levels of HO-HIF-1α following treatment with gingerol (Fig. 3E). These results suggest that the catalytic activity of PHD2 is inhibited by treatment with gingerol.

### Gingerol-induced HIF-1α expression upregulates the expression of PrPc in neurons

**Figure 4:**
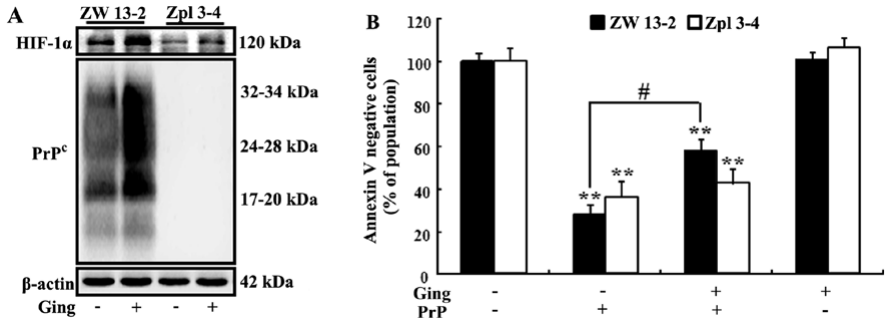

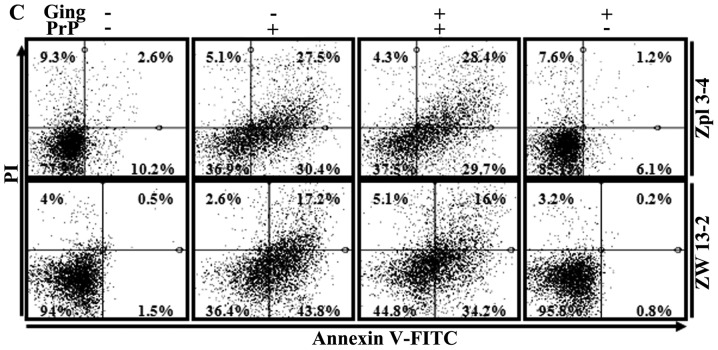
Gingerol-induced expression of hypoxia-inducible factor (HIF)-1α protein is involved in the upregulation of PrPc expression in neurons. (A) ZW 13-2 and Zpl 3–4 cells were incubated with 2.5 nM gingerol for 20 h. The expression of HIF-1α and PrPc was assessed by western blot analysis. Results were normalized to those of β-actin. (B) ZW 13-2 and Zpl 3–4 cells were incubated with 100 μM PrP (106–126) for 12 h following exposure to 2.5 nM gingerol for 12 h. Bar graph indicates the average numbers (%) of Annexin V-negative cells. ^**^P<0.01 vs. controls (untreated cells), ^#^P<0.01 vs. PrP (106–126)-treated cells. (C) Cell viability was measured by an Annexin V assay and flow cytometry. Ging, gingerol.

HIF-1α is involved in the regulation of prion protein expression to protect neurons (15). Moreover, it has also been suggested that the gingerol-induced expression of HIF-1α is a key factor in attenuating hypoxia-induced embryo toxicity (27). In our study, to determine whether HIF-1α is stabilized by gingerol-mediated PrPc expression, ZW 13-2 and Zpl 3–4 murine neuronal cells were incubated with 2.5 nM gingerol for 20 h. As shown in Fig. 4A, following treatment with gingerol, the expression of HIF-1α and PrPc was increased in the ZW 13-2 cells. However, PrPc protein expression was not detected in the Zpl 3–4 cells (Fig. 4A). To confirm the increase in the expression of PrPc following treatment with gingerol and that this caused a beneficial effect on the ZW 13-2 and Zpl 3–4 cells, the cells were incubated with PrP (106–126) 100 μM for 12 h following a 12-h exposure to 2.5 nM gingerol. Cell viability was measured by Annexin V assay and flow cytometry. In the ZW 13-2 murine neuronal cells, gingerol had a neuroprotective effect on the PrP (106–126)-induced neuronal apoptosis. In the Zpl 3–4 cells, however, it had no neuroprotective effects (Fig. 4B and C).

These results demonstrate that treatment with gingerol promotes the stabilization of HIF-1α and the expression of the HIF-1α gene. Furthermore, the gingerol-induced expression of HIF-1α increased the expression of PrPc in neurons. Taken together, these results indicate that gingerol exerts a protective effect on PrP (106–126)-induced neurotoxicity in neurons through the upregulation of HIF-1α-mediated by the expression of PrPc.

## Discussion

We conducted the present study in order to examine the effects of gingerol on PrP (106–126)-induced neurotoxicity and the interactions between gingerol and HIF-1α. Our results indicated that gingerol has therapeutic potential for use in the treatment or prevention of neurodegenerationve diseases, such as prion-related diseases.

Prion diseases involve the conversion of normal cellular prion protein into the scrapie isoform of prion protein (PrPsc) (2,14). PrPsc is widely known as an infectious agent of prion diseases (3). In addition, it is more prone to be accumulated in insoluble fibrils and thereby disturbs normal neuronal functions (2). PrP (106–126) retains the neurotoxic properties of the entire pathological PrPsc and it is generally used as a model to study the mechanisms responsible for prion diseases (12,28). However, little is known about the molecular mechanisms through which PrP (106–126)-mediated neuronal apoptosis occurs. In our previous study, we demonstrated that HIF-1α is involved in the regulation of the expression of prion protein to protect neurons (20). Furthermore, with the gingerol-induced expression of HIF-1α, human prion peptide-mediated neurotoxicity has been shown to be inhibited in neurons (15). However, little is known about the molecular mechanisms through which gingerol mediates the expression of HIF-1α. We therefore hypothesized that gingerol induces the expression of HIF-1α by inhibiting the catalytic activity of PHD2. Under normoxic conditions, HIF-1α is degraded by the hydroxylation of 2 prolines (Pro 402 and Pro 564) located within the ODD (21). In addition, HIF PHDs induce the hydroxylation of HIF-1α under normoxic conditions. Moreover, recent major advances have shown that HIF PHD2 is involved in the regulation of the ubiquitin-proteasome pathway associated with HIF-1α (3). Moreover, it is a key oxygen sensor that creates low steady-state levels of HIF-1α under normoxic conditions (3). Our results demonstrated that gingerol promoted the stabilization of HIF-1α through the inhibition of the catalytic activity of HIF PHD2 (Fig. 3). Furthermore, gingerol inhibited the hydroxylation of HIF-1α (Fig. 3A, B and E). In our study, following treatment with gingerol, there was an increase in the expression of the PHD2 gene and its products (Fig. 3C and D). These results indicated that the catalytic activity of PHD2 was inhibited as there was an increase in the gingerol-mediated expression of HIF-1α. To examine the inhibitory effects of gingerol on the catalytic activity of PHD2, we used CHX. As shown in Fig. 4E, there was a decrease in the hydroxylation of HIF-1α following treatment with gingerol in the CHX-treated group.

As shown in Figs. 1 and 2, gingerol induced the expression of HIF-1α and thereby protected the cells against PrP (106–126)-induced neurotoxicity. Moreover, it was also involved in the regulation of the catalytic activity of PHD2 by inducing the expression of HIF-1α. These results indicate that gingerol inhibits the catalytic activity of PHD2 and that this leads to the decreased hydroxylation of HIF-1α. This eventually leads to an increase in the stabilization of HIF-1α under normoxic conditions.

In a previous study, it was demonstrated that PrP (106–126)-induced neuronal apoptosis is prevented under hypoxic conditions by the activation of HIF-1α and that HIF-1α is involved in the regulation of the expression of prion protein to protect neurons (17). Furthermore, HIF-1α is involved in the regulation of the expression of PrPc in neurons (29). As shown in Fig. 4, gingerol induced the expression of HIF-1α in the ZW 13-2 murine neuronal cells, but not in the Zpl 3–4 cells. Thus, gingerol is involved in the upregulation of the expression of PrPc in ZW 13-2 neuronal cells. In the ZW 13-2 murine neuronal cells, gingerol had a neuroprotective effect on PrP (106–126)-induced neuronal apoptosis. In the Zpl 3–4 cells, however, it had no neuroprotective effects (Fig. 4B and C). These results indicate that gingerol mediates the expression of PrPc and thereby exerts a protective effect, thus, suggesting the existence of a correlation between the expression of HIF-1α and that of PrPc, both of which are induced by treatment with gingerol.

Briefly, our results demonstrated that gingerol prevented the occurrence of PrP (106–126)-induced neuronal apoptosis by upregulating the expression of PrPc. Moreover, gingerol induced the stabilization of HIF-1α by inhibiting the catalytic activity of PHD2. In conclusion, our results indicate that gingerol has therapeutic potential for use in the treatment or prevention of prion diseases by exerting inhibitory effects on the catalytic activity of PHD2, thus stabilizing HIF-1α.
